# Unpacking the dual psychological paths of employee-AI collaboration on creativity: The role of proactive behavior

**DOI:** 10.1371/journal.pone.0347335

**Published:** 2026-04-24

**Authors:** Baorong Guo, Zaifeng Wu, Quan Wen, Yifeng Peng

**Affiliations:** 1 School of Business, Guilin University of Technology, Guilin, Guangxi, China; 2 Department of Earth System Science, Tsinghua University, Beijing, China; Alexandria University Faculty of Nursing, EGYPT

## Abstract

The deep integration of artificial intelligence (AI) into organizational settings has significantly transformed employees’ work patterns, underscoring the need to investigate the mechanisms through which employee–AI collaboration influences creativity. Grounded in social cognitive theory, this study examines how employee–AI collaboration affects creativity via internal (self-efficacy) and external (performance pressure) mechanisms, and explores the moderating role of proactive behavior. Using convenience sampling, 733 valid responses were collected from six technology-driven enterprises. The theoretical model was tested through confirmatory factor analysis and regression analyses using AMOS and the Process macro for SPSS. Our results reveal that employee-AI collaboration is positively associated with creativity through two statistically supported indirect pathways: parallel indirect effects via self-efficacy (internal pathway) and performance pressure (external pathway), and a sequential indirect association in which higher self-efficacy is related to higher performance pressure, which in turn is positively related to creativity. Moreover, proactive behavior appears to moderate these relationships, such that the association between employee-AI collaboration and self-efficacy is more pronounced at higher levels of proactive behavior, whereas the association between employee-AI collaboration and performance pressure is less pronounced at higher levels of proactive behavior, yielding a pattern consistent with moderated indirect effects on creativity through both routes. These findings provide theoretical insights into the complex psychological processes underlying creativity in AI-enabled work environments and offer practical implications for optimizing AI applications and developing targeted interventions to foster employee creativity.

## 1. Introduction

AI has shifted from a speculative idea to a structuring force in the socioeconomic landscape, reshaping how work is organized and coordinated [1]. Within firms, AI’s data-processing and analytical capabilities are increasingly embedded in day-to-day processes, improving operational efficiency and decision quality while altering task interdependence and information flows [2]. Against this backdrop, employee–AI collaboration has emerged as a critical work model that materially affects employees’ behaviors and psychological states [3]. We define employee-AI collaboration as a systematic partnership in which AI and employees jointly execute tasks, participate in decision-making and prediction, solve problems, assess information, and identify opportunities and risks [4,5]. Conceptualizing collaboration in this way positions AI not merely as a tool but as a work partner whose presence reconfigures role boundaries, capability distributions, and learning opportunities.

Our focus on employee creativity follows from its status as a core source of competitive advantage and adaptive capacity for organizations. Employee creativity refers to the production of original and useful ideas for products, services, or processes [6], and it requires breaking existing mental schemas to generate novel yet feasible solutions [7]. Before the AI revolution, research identified a robust set of antecedents—including organizational climate [8], team dynamics [9], and individual traits such as psychological empowerment [10]—that reliably shape creative performance. More recently, studies situated in AI-enabled settings suggest that deeper human-AI collaboration can enhance employees’ self-efficacy and sense of meaning at work, foster psychological empowerment and job crafting, and ultimately promote innovation and creativity [11]. Related evidence links collaboration with AI to deviant innovation and AI-supported innovative behavior, indicating that AI-infused workflows may stimulate unconventional and value-creating idea generation [12,13]. These streams collectively imply that employee-AI collaboration is a possible driver of creativity.

However, these paper reflect distinct perspectives and emphasize different mechanisms. Specifically, human-computer interaction studies largely treat AI as a tool and emphasize interface/use outcomes [14], stopping short of theorizing how collaboration with AI, as a co-agent, reshapes employees’ internal and external psychological states in ways that culminate in creativity. Currently, job design studies identifies resources and demands but is mostly technology-agnostic [15]. These paper under-specifies how AI’s assistance simultaneously reduces cognitive load (internal resource) and raises performance expectations (external demand). Additionally, digital transformation studies foregrounds firm-level adoption and complementarities, but it rarely models the micro episodes of joint human–AI work [16] where creative cognition is enacted. In summary, existing mechanisms, organizational learning/task restructuring [17] and exploitative/exploratory learning at the individual level [18], illuminate pieces of the process but do not integrate the interactive psychology-context nexus of collaboration itself. We address this gap by modeling employee-AI collaboration as co-agency that activates two linked psychological pathways (self-efficacy and performance pressure) through which creativity emerges.

To this end, drawing on Social Cognitive Theory, we specify a parallel-mediation account in which employee-AI collaboration influences creativity through two psychologically distinct mechanisms. The first mechanism reflects an internal resource route: in line with Social Cognitive Theory, collaboration with AI can be seen as providing mastery experiences and diagnostic feedback that are associated with stronger self-efficacy and with a positive self-regulatory pattern that is conducive to creative action. The second mechanism reflects an external demand route: collaboration is also associated with higher evaluative visibility and expectations, which correspond to greater performance pressure and are linked to behavioral strategies oriented toward meeting higher performance standards. This duality is consistent with evidence that AI empowerment both frees cognitive resources through automation [6] and can heighten skill anxiety under perceived substitution risk [19]. Simultaneously, prior researches has identified moderators such as openness to experience affecting acceptance of AI-driven innovation [20] and job complexity shaping links between AI use and well-being [21], yet these perspectives understate how e mployees’ agentic, moment-to-moment conduct during collaboration determines what AI means for them psychologically. We therefore introduce proactive behavior as the boundary condition: proactivity—defined as a future-oriented, change-driving tendency [22]—is expected to be associated with greater mastery extraction from collaboration, for example through earlier experimentation, iterative prompting, and workflow adaptation, which aligns with a stronger association along the self-efficacy pathway. At the same time, more proactive employees are more likely to construe heightened demands as challenges rather than hindrances, which is consistent with a more tempered association along the performance pressure pathway. In this way, proactive behavior specifies the conditions under which the observed links between employee–AI collaboration and creativity, via self-efficacy and performance pressure, are most pronounced.

In summary, based on the Social Cognitive Theory, this study focuses on technology innovation-oriented enterprises to explore how employee-AI collaboration influences employee creativity through self-efficacy and performance pressure, and introduces proactive behavior as a moderating variable. Theoretically, the research model expands the research boundaries of the human-AI collaboration field by revealing the psychological mechanisms through which employee-AI collaboration stimulates creativity. Practically, the research findings will provide feasible references for management practices such as AI system design, performance management optimization, and employee behavior motivation, helping enterprises improve innovation efficiency and organizational competitiveness in the digital transformation.

## 2. Theoretical foundation and research hypotheses

### 2.1. Social cognitive theory

Social Cognitive Theory [23] emphasizes the dynamic interactive mechanisms among individual cognition, behavior, and environment, positing that external environmental stimuli shape behavioral responses by altering individual cognitive structures. The theory comprises three core modules: triadic reciprocal determinism, self-efficacy, and observational learning. In triadic reciprocity, individual cognition influences behavioral choices through processing environmental information; self-efficacy—individuals’ subjective judgment of their capabilities—regulates the efficiency of cognitive-behavioral transformation; observational learning reshapes cognitive schemas and behavioral patterns through imitation and vicarious experience acquisition [24]. These mechanisms provide a systematic theoretical tool for explaining the evolution of employee creativity in AI technological environments, while offering critical perspectives for analyzing how employee-AI collaboration influences creativity.

In recent years, scholars have illuminated the interactive mechanisms between AI and individual employee behavior from multiple dimensions based on Social Cognitive Theory. In terms of the self-efficacy pathway, empirical research shows that AI’s automated task processing and real-time feedback functions reduce employees’ cognitive load [6], enabling them to gain positive experiences of capability enhancement in complex tasks, thereby strengthening self-efficacy. When employees perceive that AI assistance can effectively address work challenges, the activity level of their creative thinking significantly increases [25]. At the observational learning mechanism level, employees can acquire data-driven innovation strategies by analyzing algorithmic decision logic during collaboration with generative AI—a form of vicarious learning that significantly promotes the diversity of creative ideation. Moreover, efficiency improvements and work model transformations brought about by observational learning from AI may intensify performance competition within organizations, leading to increased performance pressure that drives employees to continuously innovate and adapt to dynamic work environments [26]. These studies deeply embed the core elements of Social Cognitive Theory into the interactive context of employee-AI collaboration, systematically elucidating the cognitive transformation pathways through which technology enables creativity, and providing empirical evidence for organizations to optimize human-machine collaboration strategies.

### 2.2. Employee-AI collaboration and Self-efficacy

Within the systematic perspective proposed by Anthony and colleagues, employee-AI collaboration aims to accomplish work tasks through complementary strengths, thereby enhancing efficiency and quality [5,27]. As a key psychological mechanism in innovation research, self-efficacy was first proposed by Bandura (1977) and defined as an individual#39;s belief in their capability to organize and execute specific courses of action to achieve designated performance outcomes [28]. This construct emphasizes an individual#39;s subjective confidence in mobilizing existing skills to overcome challenges and achieve creative goals in specific contexts, encompassing dual dimensions of outcome attainment confidence and process control belief. In the context of organizational innovation, self-efficacy manifests as employees’ cognition and belief in their innovative capabilities, including confidence in generating novel solutions, tackling ambiguous problem spaces, and achieving innovative goals through continuous iteration [29,30].

In employee-AI collaboration, AI system provides immediate feedback and personalized support. This allows employees to promptly assess their performance and identify areas for improvement, thereby strengthening their sense of confidence and competence [31]. Additionally, AI systems decompose complex tasks into manageable steps, gradually guiding employees through the process. This reduces cognitive load and serves as an important tool for improving work efficiency and performance [32]. This step-by-step approach not only alleviates employees’ psychological pressure but also boosts their confidence when tackling complex tasks [33].

According to Social Cognitive Theory, the AI system acts as a “virtual mentor” by demonstrating efficient work processes and innovative solutions. In the employee-AI collaboration model, employees can learn new problem-solving approaches and strategies through observing and imitating AI#39;s behaviors and decision-making logic, which is likely to be associated with greater confidence in confronting difficulties and challenges, and thus with higher self-efficacy. Evidently, employee-AI collaboration is related to self-efficacy via multiple mechanisms. Therefore, the following hypothesis is proposed:

H1: Employee-AI collaboration positively influences self-efficacy.

### 2.3. The mediating role of self-efficacy

Existing studies have shown that self-efficacy is the direct driving force for employees to exhibit positive behaviors [34]. With a high level of self-efficacy, employees can more effectively mobilize resources when confronting complex work, exploring new solutions, thereby stimulating their innovative thinking and enhancing creativity. Creativity refers to the generation of novel and valuable ideas and solutions [7].Specifically, as employees’ self-efficacy increases, employees are more inclined to collaborate with AI to acquire and integrate knowledge, and to learn new approaches to task execution [35], thereby assessing necessary resources for creativity. According to Social Cognitive Theory, employees leverage AI systems to handle complex work tasks, reducing cognitive load and gaining positive experiences, and these patterns are in turn related to higher levels of self-efficacy. Therefore, the high self-efficacy formed by employees during AI collaboration may be accompanied by greater task mastery and learning motivation, and these patterns are in turn associated with higher levels of creativity. Thus:

H2: Self-efficacy mediates the relationship between employee-AI collaboration and creativity.

### 2.4. Employee-AI collaboration and performance pressure

Performance pressure is an attitudinal system referring to the perceived urgency individuals experience to achieve high-level performance [36]. Specifically, performance pressure arises when the negative consequences of failing to meet expected goals induce individuals to internalize the need for high performance, generating a sense of urgency to improve [37]. As a unique dynamic work stressor, performance pressure has been widely recognized for its significant impact on employee creativity and innovative behavior [38,39].

While the employee-AI collaboration model brings numerous conveniences, it also exposes employees to more uncertainties in career development [40]. Against the backdrop of continuous technological evolution, such uncertainties may prompt employees to maintain their career advantages by enhancing performance, thereby triggering stronger performance pressure.

Previous studies have indicated that employees’ distrust in the performance and reliability of AI systems can trigger technological insecurity, leading to concerns that AI information quality may affect work performance and further exacerbate workplace anxiety and performance pressure [41]. Moreover, employees’ over-reliance on AI systems may weaken their ability to independently solve complex problems, thereby increasing performance pressure [42].

According to Social Cognitive Theory, employees enhance work efficiency through observational learning from AI, which intensifies performance competition within organizations and consequently increases performance pressure. Therefore, the following hypothesis is proposed:

H3: The higher level of employee-AI collaboration is associated with greater perceived performance pressure.

### 2.5. The mediating role of performance pressure

As performance pressure increases, employees are more likely to appraise it as an opportunity to attain goals, satisfy needs, and create value [43]. Such appraisals foster positive evaluations of stressful events and encourage the use of approach-oriented coping strategies aimed at meeting performance standards efficiently [44]. For example, employees may seek to improve their skills and concentrate more fully on task execution, which can enhance intrinsic interest, stimulate innovative behavior [36], and, in turn, support higher levels of creativity. Empirical evidence further indicates that employees experiencing performance pressure often engage in ongoing exploratory innovation to adapt to dynamic work environments [45].

In other words, within employee-AI collaboration, employees learn AI technologies to boost work efficiency while perceiving escalated performance pressure from technological empowerment. To address this pressure, employees may be more likely to adopt innovative behaviors to break through work bottlenecks, and such behaviors are typically associated with higher levels of creativity. Therefore, the following hypothesis is proposed:

H4: Performance pressure mediates the relationship between employee-AI collaboration and creativity.

Employee–AI collaboration creates an information-rich work context that is diagnostic for the core mechanisms articulated by Social Cognitive Theory. Frequent mastery experiences (via automation and real-time feedback) [46], reduced cognitive load (through task decomposition) [47], and opportunities for vicarious learning (by observing algorithmic decision logic) jointly strengthen employees’ self-efficacy [48]. Higher self-efficacy, in turn, activates self-regulatory processes [49]: individuals elevate goal difficulty, internalize stricter performance standards, and commit to discrepancy reduction between current and desired states. In AI-mediated settings where dashboards, benchmarks, and traceable outputs render performance highly visible [50], these heightened standards become salient, making progress lapses or quality gaps more evident and personally owned.

The same self-regulatory upshift explains a positive association between self-efficacy and performance pressure. Self-efficacy does not merely reduce strain; it recalibrates appraisals by converting ambiguous demands into challenge opportunities [51]. As confidence in capability rises, the focal constraint shifts from feasibility (“can I?”) to obligation and timeliness (“I should, and swiftly”). Goal-setting and control-theoretic accounts predict that higher perceived capability elevates aspirations and narrows acceptable error margins; in data-visible, algorithmically benchmarked work systems, this produces stronger felt urgency to meet self-endorsed standards [52]. Thus, performance pressure increases not as hindrance-type distress but as challenge-type urgency that co-moves with, and is partly enabled by, elevated self-efficacy. Therefore:

H5: Employee-AI collaboration is indirectly related to employee creativity and is sequentially mediated through self-efficacy and performance pressure.

### 2.6. The moderating role of proactive behavior

Proactive behavior is defined as individuals’ intentional acts to alter situations by creating or proactively changing the existing environment [53,54]. Research shows that the satisfaction of psychological needs can significantly stimulate the emergence of employee proactive behavior [55]. Furthermore, the AMO (Ability-Motivation-Opportunity) theory constructs an influence model of employee behavior from three dimensions: ability, motivation, and opportunity. In this theory, motivation serves as the key driving force for proactive behavior.

Proactivity, as an individual trait, interacts with motivation. Employees with high proactive traits often possess stronger intrinsic motivation, making them more willing to actively learn new knowledge, enhance their capabilities, and seize work opportunities [56]. Thus, during collaboration with AI, such employees are more inclined to proactively learn AI usage and grasp its empowerment value. Conversely, employees with low proactivity may be more prone to dependence or resistance, thereby weakening the efficacy activation effect of AI.

According to Social Cognitive Theory, the extent to which employee-AI collaboration is associated with higher self-efficacy is expected to vary with employees’ own agentic characteristics. The more proactive employees are, the more likely they are to form positive feedback experiences in AI interactions, and these experiences are in turn associated with higher reported levels of self-efficacy. Therefore:

H6: Proactive behavior positively moderates the relationship between employee-AI collaboration and self-efficacy. The stronger the proactive behavior, the more obvious the promoting effect of employee-AI collaboration on self-efficacy.

We also extend the logic to the pressure pathway by tracing how proactivity alters the appraisal and regulation of demands in AI-mediated work. Employee-AI collaboration typically heightens performance pressure—defined as the perceived urgency to meet salient standards and avoid negative consequences—because rapid feedback, transparent dashboards, and fine-grained benchmarking increase evaluative visibility and compress error-correction cycles [57]. Within Social Cognitive Theory, proactive employees do not passively absorb these cues; they select and shape their environments by initiating early trials with AI tools, codifying prompts and workflow checklists, and establishing personal baselines before external comparisons intensify [58]. These anticipatory behaviors generate mastery experiences and perceived control, which narrow ambiguity at human-AI handoffs and reduce the discrepancy between current output and expected benchmarks, the immediate driver of felt urgency. AMO theory clarifies the resource mechanism: proactivity mobilizes motivation to build AI-specific abilities and to create opportunities (timely data access, peer expertise, micro-automations), thereby increasing person–task fit and slack in data-visible workflows [59]. As resources expand, identical collaboration cues are appraised less as hindrances and more as manageable, challenge-type information, converting real-time feedback into feedforward control rather than escalating pressure. Proactivity also shifts comparison frames from zero-sum social ranking to self-referenced progress metrics, further dampening evaluative threat. This moderation pertains to the direct link between collaboration intensity and pressure and coexists with the separate sequential route in which collaboration can elevate self-efficacy and, via raised aspirations, produce challenge-type urgency. Taken together, the same agentic orientation that amplifies efficacy concurrently buffers pressure growth by preempting friction, engineering coping routines, and stabilizing standards. Therefore,

H7: Proactive behavior negatively moderates the relationship between employee-AI collaboration and performance pressure. The stronger the proactive behavior, the less significant the promoting effect of employee-AI collaboration on performance pressure.

In line with the precedubg hypotheses, self-efficacy plays a mediating role between environmental stimuli and behavioral outcomes. Particularly in the context of human-machine collaboration, how individuals perceive and utilize AI technologies constitutes a key mechanism influencing their self-cognition [60]. Drawing from Social Cognitive Theory and research on proactive behavior, employees’ proactive behavior serves as a critical moderating variable, shaping their cognitive processing and experiential interpretation of AI collaboration [22].

Compared with employees low in proactivity, those with high proactive tendencies are more inclined to actively explore the application potential of AI systems in task process optimization, decision support, intelligent recommendation, etc., and proactively provide feedback and adjust usage patterns during collaboration. Such proactive exploration is likely to be associated with a stronger sense of control over AI collaboration outcomes and with the accumulation of successful experiences, which in turn are related to higher levels of self-efficacy. In contrast, low-proactivity employees may be more inclined to adopt a passive or dependent attitude and may report fewer positive feedback experiences from human–machine collaboration, patterns that are associated with lower self-efficacy. When employees’ self-efficacy is strengthened, their confidence in innovative tasks and willingness to undertake high-risk, high-creativity tasks correspondingly increase [61]. Based on this, the following hypothesis is proposed:

H8: Proactive behavior moderates the mediating role of self-efficacy between employee-AI collaboration and creativity. The stronger the proactive behavior, the stronger the positive impact of employee-AI collaboration on creativity through self-efficacy.

Building on this agency-centered account, we consider the pressure pathway. Employee–AI collaboration often elevates performance pressure by increasing performance visibility, accelerating feedback cycles, and intensifying internal comparison. Within Social Cognitive Theory, however, proactive employees shape rather than merely absorb these contingencies [62]. Through anticipatory experimentation with AI features, codification of prompts and workflow checklists, and early establishment of self-referenced baselines, they accumulate mastery experiences and perceived control prior to external evaluation. From an AMO perspective, proactivity mobilizes motivation to build AI-specific abilities and to create opportunities—timely data access, micro-automations, and peer expertise—that expand personal resources and improve person–task fit in data-visible workflows [63]. These resource gains alter primary and secondary appraisals: identical collaboration cues are less likely to be construed as evaluative threats and more likely to be treated as manageable, challenge-type information. Consequently, the incremental growth of performance pressure attributable to collaboration is attenuated, and—crucially—the degree to which that pressure translates into creativity diminishes because proactive employees rely relatively more on planned adaptation and self-regulatory routines than on urgency-driven arousal. Hence, as proactivity increases, the indirect effect of employee–AI collaboration on creativity via performance pressure weakens: collaboration generates less additional pressure, and any pressure that does arise is more efficiently regulated and less central to creative engagement. This moderated-mediation logic complements prior hypotheses by clarifying that proactivity does not uniformly amplify all indirect routes to creativity; rather, it strengthens the self-efficacy channel while buffering the pressure channel. Therefore,

H9: Proactive behavior moderates the mediating role of performance pressure in the impact of employee-AI collaboration on creativity. The stronger the proactive behavior, the weaker the positive impact of employee-AI collaboration on creativity through performance pressure.

**Fig 1:**
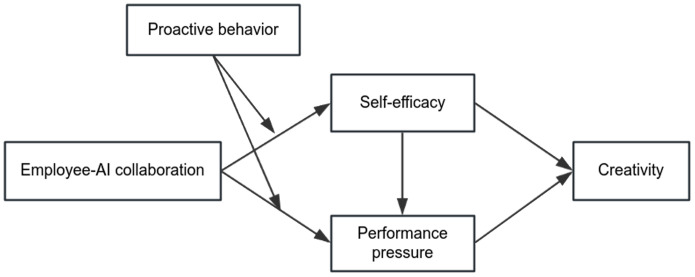
Research Model.

The research model is illustrated in Fig 1.

## 3. Methodology

### 3.1. Procedure and sample

This study adopted a questionnaire survey method to collect primary data from employees working in six technology innovation–oriented enterprises located across five provinces in China, namely Beijing, Guangdong, Fujian, Sichuan, and Zhejiang. The participating organizations operate in industries such as information technology, electronics, telecommunications, and software development. The questionnaire and overall research protocol were reviewed and approved by the ethics committee of Guilin University of Technology (Ethics approval number: 202406000202). Before launching the survey, the research team contacted senior managers and Human Resources (HR) representatives in each enterprise, explained the research objectives, the content of the questionnaire, and the data protection measures, and obtained organizational consent on the basis that participation would be voluntary, responses would remain anonymous, and only aggregated results would be reported back to the companies.

After organizational approval, HR departments in the six enterprises distributed QR codes linked to the online questionnaire via internal communication channels. Employees were informed that the study concerned their experiences of working with AI in their daily tasks and that there were no right or wrong answers. They could access the questionnaire using their personal devices (e.g., smartphones or computers) and complete it at a time and place of their choosing, outside of direct managerial supervision. The introduction page of the survey reiterated that participation was entirely voluntary, that respondents could discontinue at any time without any consequences, and that no identifying information such as names or employee ID numbers would be collected. Only respondents who provided informed consent proceeded to the substantive items. Data collection took place between 1 June 2024 and 1 September 2025 and, for practical reasons, was implemented in two closely spaced phases within this overall window.

In the first phase of data collection (1 June to 3 September 2024), HR distributed the survey link and QR codes to eligible employees, and 523 questionnaires were returned. Using pre-specified quality-control criteria, we excluded questionnaires with uniform response patterns suggesting a lack of engagement (e.g., the same option selected for all items), or with completion times far below a reasonable minimum (180 seconds) for thoughtful responding. On this basis, 22 cases were removed, resulting in 501 valid responses. These 501 responses formed the empirical basis for the analyses reported in the first version of the manuscript. Although all focal constructs were measured using well-established multi-item scales widely applied in high-level research, some reliability and validity indices in the initial analyses were at the lower bound of recommended thresholds for complex models combining multiple latent variables, parallel and sequential mediation, and moderated mediation. Given the theoretical importance of retaining the full model and the scales, we considered it more appropriate from a scientific standpoint to increase the sample size within the same research context rather than to simplify the model or drop constructs solely for statistical reasons.

Accordingly, we conducted a second phase of data collection in the same six enterprises, within the 8 August to 1 September 2025 period, using the identical questionnaire, sampling frame, recruitment channels, and ethical safeguards. HR departments again circulated the same QR codes to employees who met the inclusion criteria, and the survey remained voluntary and anonymous under the same conditions as in Phase 1. In this second phase, 277 additional questionnaires were returned. Applying exactly the same screening criteria as before (missing data, response patterns, and completion time), 45 questionnaires were excluded, yielding 232 further valid responses. In other words, the two phases constitute one continuous cross-sectional survey effort administered in two administrative rounds, with no change in companies, measurement instruments, or survey procedures.

For the present version of the paper, we pooled the data from the two phases into a single dataset and treated them as originating from the same underlying population of employees working in technology innovation–oriented enterprises. Across both phases, a total of 800 questionnaires were received. After excluding 67 invalid questionnaires (22 from the first phase and 45 from the second), the final analytical sample comprised 733 valid responses. To assess the comparability of the original 501 cases and the additional 232 cases, we compared the distributions of key demographic variables (gender, age, education, tenure, and position) and the descriptive statistics of the focal constructs (employee–AI collaboration, proactive behavior, self-efficacy, performance pressure, and creativity) across the two subsamples. These comparisons indicated highly similar patterns and no systematic differences in either demographic composition or mean levels of the study variables, supporting the view that the two phases represent repeated draws from the same organizational settings under identical conditions. On this basis, all reliability, validity, and hypothesis tests reported in the Results section are based on the combined sample of 733 respondents; the earlier 501-case dataset was used only for preliminary model estimation in the first manuscript version and no longer serves as the empirical basis for the conclusions presented here.

Among the 733 respondents in the final pooled sample, men accounted for 50.9% and women for 49.1%. Employees aged 21–30 accounted for 39.0%, those aged 31–40 for 34.1%, and those over 41 years old for 26.9%. With respect to education, 34.9% held a junior college degree or below, 37.2% held a bachelor’s degree, and 27.8% held a postgraduate degree or above. In terms of organizational tenure, 25.8% of the employees had worked for less than 2 years, 36.0% had worked for 3–5 years, 21.0% had worked for 6–10 years, and 17.2% had worked for more than 11 years. Regarding job position, 14.7% were in management roles, 26.1% in technical positions, 22.0% in R&D positions, 23.6% in service roles, and 13.6% in other positions. Overall, the sample thus covers a broad range of demographic backgrounds and job roles within technology innovation–oriented enterprises, providing a robust empirical foundation for examining the relationships among employee–AI collaboration, self-efficacy, performance pressure, proactive behavior, and creativity.

### 3.2. Measurement

Drawing on the propositions of existing scholars [64], this study adopts well-established scales with good reliability and validity. The English scales were translated into Chinese following the standard double-blind back-translation procedure, with linguistic adjustment for this specific topic. All items are rated on a 5-point Likert scale ranging from 1 (strongly disagree) to 5 (strongly agree) unless explained separately.

Employee-AI Collaboration: The employee-AI collaboration is measured with the scale developed by Kong et al. [4], which consists of 5 items. One sample is “AI participates in my decision-making process.”

Proactive behavior: The single-dimensional scale of employee proactive behavior is developed by Frese et al. [65], which includes a total of 7 items. One sample is “Usually, I do more work than what my supervisor requires.”

Self-efficacy: Self-efficacy is measured using the 10-item scale developed by Zhang & Schwarzer [66]. One sample is “With my intelligence, I can definitely deal with unexpected situations.”

Performance Pressure: Performance pressure is measured using the 4-item scale developed by Mitchell et al. [67], One sample is “The performance pressure in my workplace is very high.”

Creativity: Employee creativity is measured using the 4-item developed by Farmer et al. [68], which was originally designed for Chinese employees. One sample is “I take the initiative to try out new ideas or methods.”

Control Variables. Demographic variables such as gender (0 = male; 1 = female), age (1 = 21–30 years; 2 = 31–40 years; 3 = 41 + years), education level (1 = junior college or below; 2 = bachelor’s degree; 3 = postgraduate or above), and work experience (1 = < 2 years; 2 = 3–5 years; 3 = 6–10 years; 4 = > 11 years) were treated as control variables, as previous research has shown they can affect creativity [69].

## 4. Results

### 4.1. Reliability and validity

The factor loadings, Cronbach’s alpha coefficients, composite reliability (CR), and average variance extracted (AVE) values for each construct are reported in Table 1.

**Table 1:** Reliability and convergent validity tests.

Variables	Loadings	Cronbach’s α	CR	AVE
Employee-AI collaboration	0.655 ~ 0.743	0.849	0.818	0.474
Proactive behavior	0.654 ~ 0.731	0.874	0.860	0.468
Self – efficacy	0.611 ~ 0.715	0.912	0.891	0.451
Performance pressure	0.682 ~ 0.772	0.839	0.826	0.544
Creativity	0.662 ~ 0.796	0.832	0.813	0.523

Note: N = 733, and the same applies hereinafter.

In interpreting these psychometric indicators, it is important to clarify the empirical basis on which they are estimated. At an early stage of the project, preliminary analyses were conducted on 501 valid responses obtained from an initial round of data collection in the six participating enterprises. To enhance the precision and stability of the measurement and structural models, data collection was subsequently extended within the same organizational settings, using the identical questionnaire, HR-mediated QR-code distribution procedure, and inclusion criteria. This second round yielded a further 232 valid responses after applying the same exclusion rules for missing data, low-quality response patterns, and unrealistically short completion times. Because both rounds shared the same companies, sampling frame, and protocol, they represent two administrative phases of a single cross-sectional survey. We therefore pooled the 501 and 232 cases into one dataset of 733 employees and used this full sample for all reliability and validity tests, as well as for the subsequent regression, mediation, and moderated-mediation analyses. Comparisons of demographic characteristics and mean scores on the focal constructs between the two subsamples indicated no systematic differences, supporting the view that they are repeated draws from the same population under equivalent survey conditions and that the psychometric evidence reported in Tables 1, 2 rests on a coherent and scientifically rigorous empirical foundation.

**Table 2:** Confirmatory Factor ANalysis.

Models	Χ^2^	df	X^2^/df	RMSEA	CFI	TLI	GFI
Six-factor model	515.277	365	1.412	0.024	0.986	0.984	0.956
Five-factor model	697.279	395	1.765	0.032	0.972	0.970	0.940
Four-factor model	1523.973	399	3.819	0.062	0.898	0.888	0.844
Three-factor model	2250.357	402	5.598	0.079	0.832	0.818	0.761
Two-factor model	2746.400	404	6.798	0.089	0.787	0.770	0.718
Single-factor model	3204.942	405	7.913	0.097	0.745	0.726	0.685

Confirmatory factor analysis (CFA) was conducted using AMOS 26 and SPSS 27 to test the discriminant validity among variables. Four competing models were test, in which the four-factor model combined self-efficacy and performance pressure into one factor, the three-factor model merged proactive behavior, self-efficacy and performance pressure into one factor, the two-factor model grouped employee-AI collaboration, proactive behavior, self-efficacy and performance pressure into one factor, and the single-factor model integrated all variables into one factor. The analysis results are shown in Table 2. It can be seen that the fit indices of the hypothesized five-factor model all meet the standards and are superior to those of other competing models, indicating good discriminant validity among the variables.

### 4.2. Common method bias test

In this paper, Harman#39;s single-factor test is employed to assess common method bias. Exploratory factor analysis is conducted on all the measurement items across the five variables using SPSS. The results show that the first component explains 39.24% of the total variance, which is lower than the judgment criterion of 40%. In addition, to address the limitation of relying solely on Harman’s single-factor test, we conducted a latent common-method factor test by adding a common-method factor to the original five-factor measurement model. As reported in S2 Table, this alternative specification resulted in only trivial changes in model fit relative to the original measurement model, with ΔRMSEA = 0.008, ΔCFI = 0.014, ΔGFI = 0.014, and ΔTLI = 0.016. Since these changes were all below 0.02, the latent method factor did not meaningfully improve model fit, suggesting that shared method variance is unlikely to have substantially biased the observed relationships. This pattern is consistent with the confirmatory factor analysis results in Table 2 and provides further support for the robustness of the measurement model.

### 4.3. Descriptive statistics and correlation analysis

Table 3 shows the means, standard deviations and the correlation coefficients of the variables. As shown in Table 3, employee-AI collaboration has a significant positive correlation with creativity (r = 0.560, p < 0.01), self-efficacy (r = 0.607, p < 0.01), and performance pressure (r = 0.550, p < 0.01). Self-efficacy has a significant positive correlation with creativity (r = 0.522, p < 0.01), and performance pressure has a significant positive correlation with creativity (r = 0.536, p < 0.01), providinh preliminary support for the hypothesis testing.

**Table 3:** Descriptive statistics and correlation analysis.

Variables	Mean	SD	1	2	3	4
1.Employee-AI collaboration	3.369	0.992	1			
2.Proactive behavior	3.334	0.902	0.524^**^	1		
3.Self – efficacy	3.368	0.912	0.607^**^	0.610^**^	1	
4.Performance pressure	3.335	0.982	0.550^**^	0.509^**^	0.475^**^	1
5.Creativity	3.345	0.979	0.560^**^	0.465^**^	0.522^**^	0.536^**^

Note: *p < 0.05; **p < 0.01; ***p < 0.001. The same applies hereinafter.

### 4.4. Hypothesis testing

Hypothesis testing was conducted using a unified conditional process framework based on regression analysis and implemented with PROCESS. We utilised a parallel multiple mediation model (Model 4) incorporating both self-efficacy and performance pressure to simultaneously estimate the two indirect effects corresponding to H2 and H4 within a single model. Subsequently, a sequential mediation model (Model 6) examined the sequential indirect association from self-efficacy to performance pressure (Hypothesis 5). Finally, a first-stage moderated mediation model (Model 7) examined whether proactive behaviour moderates the association between employee-AI collaboration and each mediating variable (Hypotheses 8–9). Indirect effects were assessed using 5,000 guided resampling and 95% bias-corrected confidence intervals. Gender, age, educational attainment, tenure, and job position were included as covariates in both the mediating variable and outcome equations. Continuous variables involved in interaction terms underwent mean centring prior to analysis. As the questionnaire employed a mandatory response format, no item-level missing data existed.

Previous studies have shown that employees’ perceptions and intergrations of AI collaboration are primarily reflected at the individual level [4]. Moreover, due to the high complexity and heterogeneity of AI technology, significant individual differences exist in employees’ performance during AI-human collaboration [54]. Therefore, following the common practice in existing research, this study employs an individual-level aproach method to test the research hypotheses [70]. The results are presented in Table 4.

**Table 4:** Path analysis table.

Variables	Creativity		Self – efficacy		Performance pressure	
	β	SE	β	SE	β	SE
Gender	0.041	0.055	−0.039	0.048	−0.026	0.055
Age	−0.030	0.035	−0.064^*^	0.030	0.046	0.034
Education	−0.036	0.035	−0.004	0.030	0.032	0.035
Service tenure	−0.010	0.027	−0.020	0.023	0.020	0.027
Position	0.042	0.022	0.032	0.019	0.045^*^	0.022
Employee-AI collaboration	0.271^***^	0.038	0.391^***^	0.029	0.334^***^	0.033
Proactive behavior			0.423^***^	0.031	0.306^***^	0.036
Interaction term			0.116^***^	0.025	−0.249^***^	0.029
Self – efficacy	0.236^***^	0.039			0.244^***^	0.041
Performance pressure	0.280^***^	0.035				
R^2^	0.417		0.502		0.428	

Mediation Analysis of Self-Efficacy. As shown in Table 4, employee-AI collaboration has a positively effect on self-efficacy (β = 0.391, SE = 0.029, p < 0.001), thus supporting H1. Furthermore, self-efficacy also positively affects creativity (β = 0.236, SE = 0.039, p < 0.001). According to the Bootstrap test results in Table 5, the mediation effect of self-efficacy in the relationship between employee-AI collaboration and creativity is 0.132, with a 95% confidence interval of [0.082, 0.188]. Since this interval does not include zero, it indicates a significant mediating effect of self-efficacy, thus supporting H2.

**Table 5:** Mediation Effect Test.

Path	β	SE	95%CI	
			LB	UB
Employee-AI collaboration→Self-efficacy→Creativity	0.132	0.027	0.082	0.188
Employee-AI collaboration→Performance pressure→Creativity	0.152	0.023	0.108	0.198
Employee-AI collaboration→Self-efficacy→Performance pressure→Creativity	0.039	0.010	0.021	0.060

Mediation Analysis of Performance Pressure. Employee-AI collaboration has a positive effect on performance pressure (β = 0.334, SE = 0.033, p < 0.001), and performance pressure further positively influences creativity (β = 0.280, SE = 0.035, p < 0.001). This validates H3 and provides initial evidence for H4. The mediation effect of performance pressure in the relationship between employee-AI collaboration and creativity is 0.152, with a 95% confidence interval of [0.108, 0.198]. Since this interval does not include zero, it indicates a significant mediation effect of performance pressure, thus supporting H4.

Based on the analysis results in Table 4, employee-AI collaboration exerts a positive impact on self-efficacy (β = 0.391, SE = 0.029, p < 0.001), while self-efficacy has a positive influence on performance pressure (β = 0.244, SE = 0.041, p < 0.001), and performance pressure can positively affect employees’ creativity (β = 0.280, SE = 0.035, p < 0.001). Meanwhile, the results in Table 5 indicate a statistically significant sequential indirect association from employee-AI collaboration to employees’ creativity via self-efficacy and performance pressure (β = 0.039, 95% CI = [0.021, 0.060]), which is consistent with the hypothesized pattern in H5.

**Fig 2:**
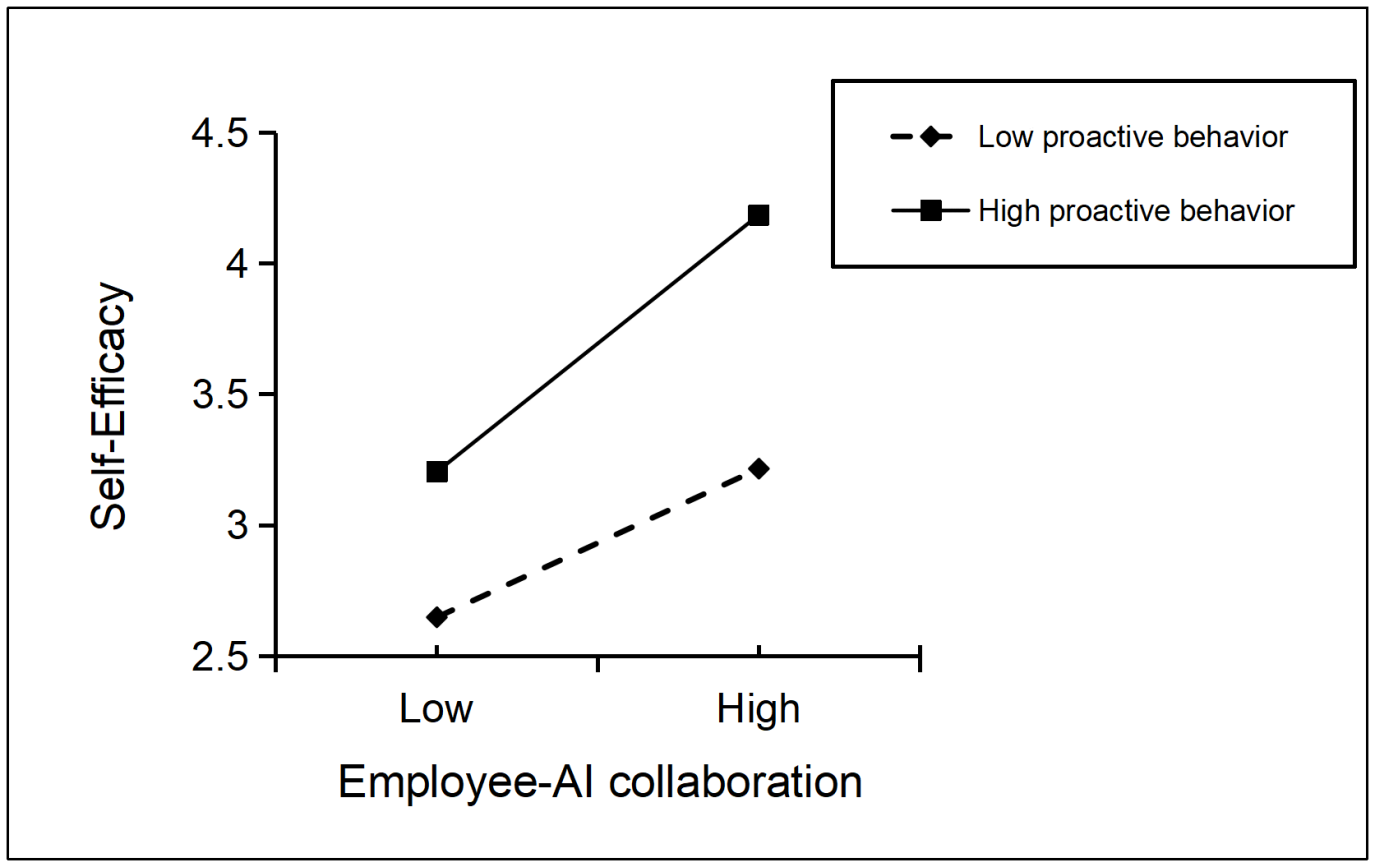
Moderating Effect Diagram of Proactive Behavior on the Relationship between Employee-AI Collaboration and Self-Efficacy.

Moderating Analysis of Proactive Behavior. When controlling for the interaction term, the interaction term between employee-AI collaboration and proactive behavior has a positive effect on self-efficacy (β = 0.116, SE = 0.025, p < 0.001). To better illustrate the moderating effect of proactive behavior, a moderating effect graph of the influence of employee-AI collaboration on self-efficacy under high-level (Mean + 1SD) and low-level (Mean – 1SD) proactive behavior was drawn, as shown in Fig 2. As can be seen from Fig 2, the positive impact of employee-AI collaboration on self-efficacy is stronger when proactive behavior is at a high level (β = 0.495, SE = 0.039, p < 0.001) than when it is at a low level (β = 0.286, SE = 0.033, p < 0.001), thus H6 is supported.

**Fig 3:**
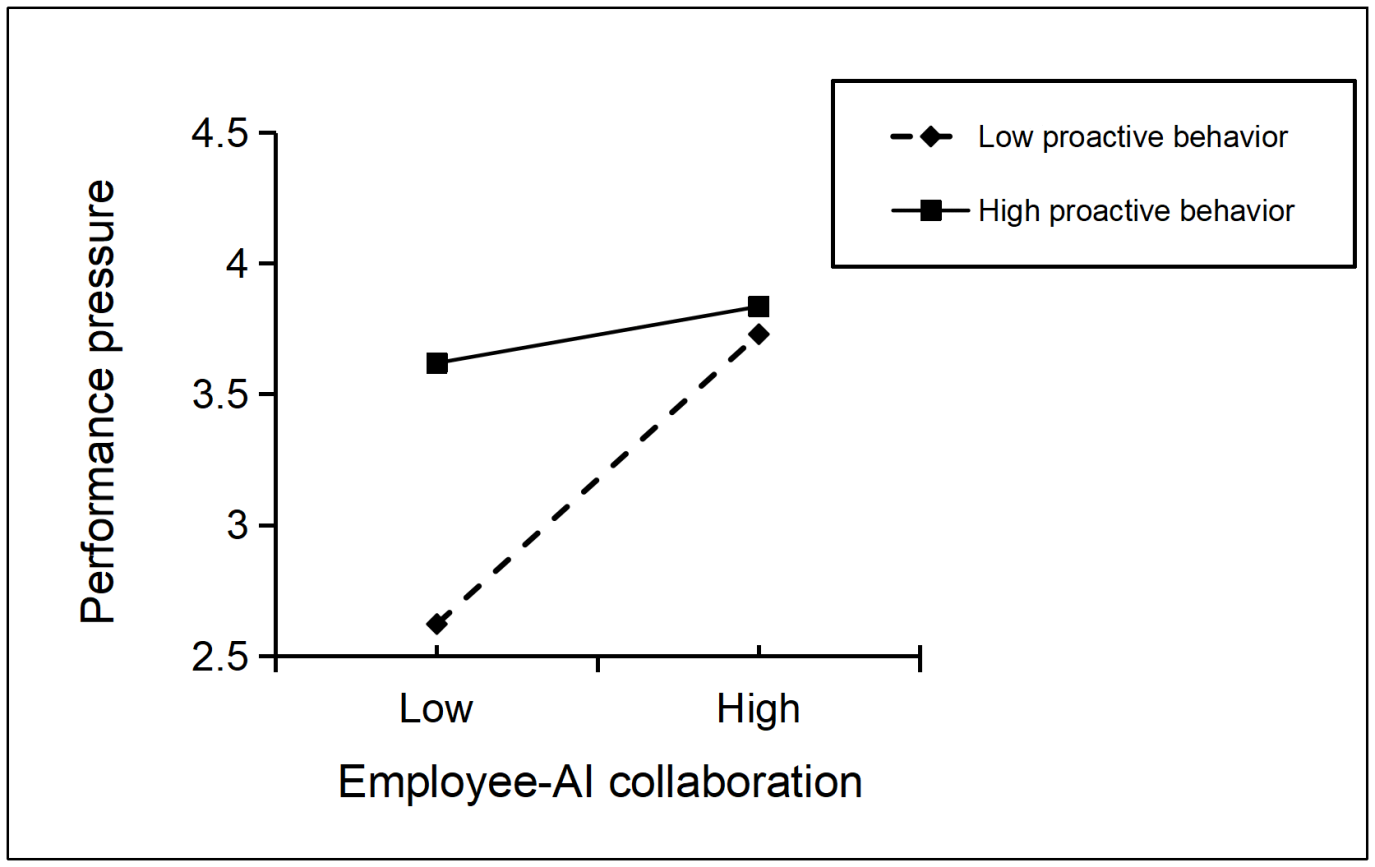
Moderating Effect Diagram of Proactive Behavior on the Relationship between Employee-AI Collaboration and Performance Pressure.

Similarly, the interaction term between employee-AI collaboration and proactive behavior has a negative impact on performance pressure (β = −0.249, SE = 0.029, p < 0.001). As shown in the moderating effect in Fig 3, the impact of employee-AI collaboration on performance pressure is significantly stronger when proactive behavior is at a low level (β = 0.558, SE = 0.038, p < 0.001) than when it is at a high level (β = 0.109, SE = 0.046, p = 0.017), thus supporting H7.

Moderated Mediation Analysis. Table 6 presents the results of the moderated mediation analysis. As shown in Table 6, the model indicates a statistically significant indirect effect of employee-AI collaboration on employee creativity via self-efficacy when proactive behavior is high (β = 0.117, 95% CI = [0.072, 0.166]), and this indirect effect is stronger than when proactive behavior is low (β = 0.068, 95% CI = [0.035, 0.112]). The difference in the indirect effect between high and low levels of proactive behavior is 0.049, with a 95% CI of [0.022, 0.080], indicating a significant difference. These results suggest that proactive behavior is positively associated with the strength of the indirect relationship between employee-AI collaboration and creativity via self-efficacy, and are consistent with H8. Furthermore, for the path involving performance pressure, the indirect effect of employee-AI collaboration on creativity via performance pressure is statistically significant when proactive behavior is high (β = 0.031, 95% CI = [0.005, 0.061]), and is even more pronounced when proactive behavior is low (β = 0.156, 95% CI = [0.113, 0.203]). The difference in the indirect effect between high and low levels of proactive behavior is −0.125, with a 95% CI of [−0.168, −0.086], indicating a significant difference. These findings indicate that proactive behavior is associated with a weaker indirect relationship between employee-AI collaboration and creativity via performance pressure at higher levels of proactivity, thereby supporting H9.

**Table 6:** The test of the moderated mediation effect.

Moderator variable	Employee-AI collaboration→Self-efficacy→Creativity			Employee-AI collaboration→Performance pressure→Creativity		
	β	SE	95%CI	β	SE	95%CI
Mean+1SD	0.117	0.024	[0.072,0.166]	0.031	0.014	[0.005,0.061]
Mean-1SD	0.068	0.020	[0.035,0.112]	0.156	0.023	[0.113,0.203]
Difference	0.049	0.015	[0.022,0.080]	−0.125	0.021	[-0.168,-0.086]

### 4.5. Robustness test

To test the robustness of the research results, following the approach of existing studies [71], a test on the sample data is conducted without including control variables. This result showed that, even when excluding the control variables, employee-AI collaboration continues to exert a significant positive effect on self-efficacy (β = 0.388, SE = 0.029, p < 0.001) and performance pressure (β = 0.335, SE = 0.033, p < 0.001). Furthermore, self-efficacy (β = 0.135, 95% CI = [0.084, 0.194]) and performance pressure (β = 0.152, 95% CI = [0.109, 0.200]) remains stable mediator between employee-AI collaboration and employee creativity.

In addition, the interaction term between employee-AI collaboration and proactive behavior continue moderate the influence of employee-AI collaboration on self-efficacy (β = 0.117, SE = 0.025, p < 0.001) and performance pressure (β = −0.247, SE = 0.029, p < 0.001). Table 7 summarizes the results of moderated mediation effect conducted without control variables. The mediating effect of employee-AI collaboration on employee creativity through self-efficacy is stronger under high levels of proactive behavior (β = 0.119, 95% CI = [0.074, 0.170]) than under low levels of proactive behavior (β = 0.069, 95% CI = [0.035, 0.115]). Similarly, the mediating role of employee-AI collaboration in influencing employee creativity through performance pressure is lower under high-level proactive behavior (β = 0.032, 95% CI = [0.006, 0.062]) than under low-level proactive behavior (β = 0.156, 95% CI = [0.113, 0.205]).

**Table 7:** The Test of the Moderated Mediation Effect without Control Variables.

Moderator variable	Employee-AI collaboration→Self-efficacy→Creativity			Employee-AI collaboration→Performance pressure→Creativity		
	β	SE	95%CI	β	SE	95%CI
Mean+1SD	0.119	0.025	[0.074,0.170]	0.032	0.014	[0.006,0.062]
Mean-1SD	0.069	0.020	[0.035,0.115]	0.156	0.023	[0.113,0.205]
Difference	0.050	0.015	[0.021,0.080]	−0.124	0.021	[-0.168,-0.087]

Furthermore, to rule out any potential influence of differences between the two data collection phases on the core findings, we conducted independent hypothesis tests on the data from the second phase. The results indicate that both the direction and significance of the effects of the core variables remained consistent, suggesting that phase differences did not drive the key findings of this study. These test results collectively validate the reliability of the pooled analysis and the robustness of the conclusions. For detailed analysis results, please refer to the Supporting Information.

## 5. Discussion

### 5.1. Theoretical contributions

Firstly, against the backdrop of enterprise digital transformation, our findings highlight employee-AI collaboration as a novel antecedent of employee creativity. This expands the creativity literature beyond traditional drivers like organizational climate [72], incentive systems [73], or leadership style [74], which have dominated prior research. In contrast to studies focusing on those human factors, we position AI as an environmental resource that dynamically shapes employee cognition and behavior. Notably, we demonstrated that engaging collaboratively with AI can boost employees’ creative output by reshaping their psychological states. This addresses a key gap – organizations have struggled to predict how AI integration influences innovation. As recent work observes, the rapid adoption of AI is “profoundly altering the workplace” and calls for elucidating its impact on employee innovation [20]. Our study directly responds to this call by showing that AI tools are not just static aids (as traditional HCI theories imply) but active elements of the work environment that can elevate an employee’s creative potential via changes in their self-perception and pressures. By moving beyond a purely instrumental view of AI, we provide evidence that employee-AI collaboration can serve as a catalyst for creativity through its influence on employees’ mindset and motivation, thereby helping bridge the disconnection between AI applications and existing work patterns. This enriches the theoretical discourse on digital-era creativity and offers a fresh lens for understanding innovation in AI-enabled workplaces.

Secondly, this study deepens theoretical explanations of the internal mechanisms linking AI collaboration to creativity by examining the dual mediating roles of self-efficacy and performance pressure. Prior studies have often isolated single factors (e.g., only motivation or only stress) when explaining employee creativity [75], and our parallel mediation analysis revealed two distinct psychological pathways through which employee-AI collaboration is associated with creativity in this cross-sectional study. On one hand, our finding that AI usage raises self-efficacy and thereby innovation aligns with recent empirical evidence, reinforcing the idea that AI can function as a “digital co-worker” that strengthens personal agency [76]. On the other hand, we found that AI collaboration can also heighten perceived performance pressure – likely because efficiency gains from AI lead managers and employees to set more ambitious goals and expectations. Importantly, rather than hampering creativity, this increased pressure in our context also drove employees to be more creative. This suggests that the pressure was interpreted as a challenge to meet higher standards, motivating greater creative effort (consistent with the challenge-stressor perspective). These two synergistic pathways – one amplifying personal capability (self-efficacy) and the other intensifying performance demands – work in tandem to explain how AI collaboration translates into creative outcomes. By integrating both a self-efficacy path and a performance pressure path, we construct a more comprehensive framework for understanding digital technology–enabled creativity. This dual-path model extends Social Cognitive Theory’s application to human–AI contexts, illustrating how environmental technology can simultaneously provide empowering experiences and introduce new pressures, both of which reciprocally influence creative behavior. Building on this theoretical extension, our approach thereby responds to calls for more systematic exploration of multiple mediators in creativity research and underscores the complex, bidirectional nature of cognitive and environmental interactions in the digital era [77].

Third, this study introduces proactive behavior as a key moderating variable, adding an individual-differences dimension to the employee–AI collaboration framework. Prior creativity research has largely emphasized external enablers or psychological states (e.g., climate, support, intrinsic motivation) [78,79], whereas much less is known about how personal traits interact with advanced technologies to shape creative performance. By incorporating proactive behavior—a self-initiated, change-oriented tendency—as a core individual characteristic, we respond to recent calls to examine how dispositional factors modulate technology’s impact on creativity [77]. Our moderation results indicate that proactive behavior systematically conditions how the technological environment relates to employees’ psychological resources. In particular, proactive employees appear to experience AI collaboration differently from their less proactive counterparts. Proactive behavior strengthens the positive association between employee–AI collaboration and self-efficacy, consistent with Social Cognitive Theory’s emphasis on mastery experiences and agentic self-regulation [80]. Employees high in proactivity are more likely to actively appropriate AI capabilities—experimenting earlier, iterating prompts and workflows, and seeking diagnostic feedback—thereby turning collaboration episodes into richer mastery experiences and clearer performance schemas. This agentic engagement enhances perceived controllability at human–AI handoffs, accelerates learning cycles, and reinforces efficacy beliefs more strongly than for less proactive peers. In this sense, proactivity and AI collaboration are complementary: proactivity supplies initiative and exploratory effort, while AI provides fine-grained feedback and scalable assistance, and together they amplify gains in self-efficacy.

At the same time, our results confirmed that proactive behavior moderates the performance pressure pathway in line with our predictions (H7). Employees high in proactive behavior felt significantly less performance pressure from AI collaboration compared to those low in proactivity. This finding dovetails with prior evidence that proactive employees cope better with job demands – they tend to experience lower strain under challenging conditions [81]. In practice, proactive individuals likely anticipate challenges and actively manage their tasks, preventing AI-enabled efficiency gains from overwhelming them. By contrast, less proactive employees may be more vulnerable to feeling stressed or pressured when work expectations rise in an AI-supported environment. Consequently, proactive behavior buffered the potentially negative side-effects of AI, ensuring that increased efficiency did not translate into debilitating pressure. Moreover, we found that proactive behavior’s moderating influence carries through to creative outcomes (H8 and H9): the indirect positive association between AI collaboration and creativity via self-efficacy is stronger at higher levels of proactive behavior, whereas the indirect association via performance pressure is weaker at higher levels of proactive behavior. In combination, these moderated mediation results mean that employees with a proactive orientation ultimately realized greater creative benefits from AI collaboration than those who are passive. This resonates with recent field evidence that simply implementing AI tools is not enough – how employees engage with the AI is critical [82]. For instance, existing research found that generative AI only boosted creativity for employees who actively reflected on and adapted their use of the tool [83]. This underscores that human agency remains a decisive factor in leveraging AI for innovation. This contribution enriches the creativity literature by bridging technological and individual-level factors. It answers calls in the literature to integrate personal behavioral traits into models of tech-enabled creativity, and provides a more nuanced theoretical account of why the same AI tools might spur high creativity in some individuals but not in others [77]. In summary, the inclusion of proactive behavior as a moderator refines our understanding of the human–AI collaboration process, revealing that the benefits of AI for creativity are maximized when employees take an active, initiative-driven role, whereas a lack of proactivity can diminish those benefits or exacerbate pressures.

### 5.2. Practical contributions

First, building on our dual-pathway model, we suggest that organizations reposition AI from a passive tool to an active co-agent in the creative process. This shift requires HR to lead structured capability-building initiatives that strengthen employees’ creative self-efficacy and help them construe performance pressure as motivating rather than hindering. Proactive behavior should be cultivated as a key boundary condition under which collaboration with AI is most strongly associated with creative gains. To support this, organizations need to redesign training and performance management systems so that employees regularly engage in mastery experiences with AI, receive rapid feedback on progressively more complex tasks, and are encouraged to experiment and refine workflows without fear of punitive responses to thoughtful failure. An HR-led “AI–Creativity Accelerator” program can be used to translate these principles into practice and foster more effective creative collaboration.

Second, the “AI–Creativity Accelerator” program can be implemented as a cohort-based initiative over six to eight weeks, with brief workshops embedded into employees’ ongoing projects. The initial phase should focus on decomposing complex tasks and generating “quick wins” with AI, enabling employees to experience competence gains and vicarious learning that reinforce creative self-efficacy. As the program progresses, later sessions can introduce stretch goals and time-limited challenges that frame performance expectations as challenge-oriented pressure, supported by regular, formative feedback from managers. Throughout the program, participants are encouraged to identify opportunities, experiment with AI prompts and configurations, and document workflow improvements, thereby fostering proactive behavior in real work settings. Evaluation should combine pre- and post-measures of creative self-efficacy and challenge versus hindrance appraisals with concrete performance indicators such as implemented ideas, time-to-prototype, and error rates, ensuring that psychological gains are reflected in measurable outcomes.

Third, industry contexts can adapt these principles by tailoring them to domain-specific tasks. In software development, HR can collaborate with engineering teams to design paired sessions in which developers rotate roles (e.g., driver, reviewer, prompt architect) while using AI-assisted coding tools to refactor legacy modules, with success assessed through accepted design improvements and reduced bug-fix cycle times. In financial services and compliance, analysts can use AI to draft risk reports and derive novel risk signals from AI model rationales, with performance tracked through review times and the adoption rate of new risk indicators. This flexibility in application illustrates how the proposed model can be adapted to drive creative innovation across diverse sectors.

### 5.3. Research limitations and future research directions

Despite its contributions, this study has several limitations that point to avenues for future research. First, the sample was drawn from six technology-innovation-oriented enterprises located in leading regions of AI development in China (e.g., Beijing and Guangdong). Although these firms capture key features of China’s innovation-driven enterprises, the extent to which our conclusions generalize to more traditional industries remains unclear. Future research could broaden the sampling frame and conduct comparative analyses across sectors—for example, manufacturing and service industries—to examine whether industry characteristics moderate the relationships specified in the theoretical model.

Second, the cross-sectional research design restricts the ability to draw strong causal inferences about the associations among employee–AI collaboration, self-efficacy, performance pressure, and creativity. While the proposed model is grounded in established theory and supported by robust statistical analyses, longitudinal or experimental designs would provide stronger evidence regarding causal direction. Future studies could adopt time-lagged or multi-wave designs to trace the dynamic evolution of employee perceptions and behaviors in AI-augmented work environments and to validate the temporal ordering of the mediating and moderating mechanisms proposed in this study.

## 6. Conclusion

This study, grounded in the Social Cognitive Theory, took employees collaborating with artificial intelligence in technology innovation-oriented enterprises as the research sample. Through the analysis of 733 questionnaire datasets, this study suggests that self-efficacy and performance pressure may play mediating roles in the association between employee-AI collaboration and creativity, providing the necessary confidence and motivation for stimulating creativity. This enables employees to break through traditional thinking patterns and actively explore new approaches to address challenges, thus promoting creative output.

Notably, proactive behavior acts as a key moderating variable in this complex relational network, regulating not only the direct relationship between employee-AI collaboration and self-efficacy but also the indirect pathway through which collaboration influences creativity via self-efficacy. Specifically, when the level of collaboration is low, employees with high proactive behavior demonstrate significantly higher self-efficacy, as they can enhance work effectiveness by tapping into personal potential and proactively acquiring resources. At higher levels of employee-AI collaboration, the positive association with self-efficacy is stronger when proactive behavior is lower, indicating a compensatory pattern and a negative collaboration-by-proactive behavior interaction on self-efficacy. In contrast, self-efficacy enhancement among high-proactivity employees tends to stabilize, potentially due to the higher expectations and self-imposed pressure during collaboration.

These findings highlight the critical role of individual differences in determining the effectiveness of human-AI collaboration in digital work settings. The study emphasizes that enterprises should formulate customized strategies to maximize the value of employee-AI collaboration and facilitate the cultivation and stimulation of employee creativity.

## Supporting information

S1 Table Measurement Items. (DOCX)

S2 Table The Latent Common-Method Factor Test. (DOCX)

S3 Table Descriptive Analysis of Phase Two Samples. (DOCX)

S4 Table Data Path Analysis for Phase Two. (DOCX)

S5 Table The Mediating Effect for Second Phase. (DOCX)

S6 Table The Moderated Mediating Effect Test for Second Phase. (DOCX)
