# Correction: Unpacking the dual psychological paths of employee-AI collaboration on creativity: The role of proactive behavior

**DOI:** 10.1371/journal.pone.0350758

**Published:** 2026-06-01

**Authors:** Baorong Guo, Zaifeng Wu, Quan Wen, Yifeng Peng

The Funding statement for this article is incorrect. The correct Funding statement is as follows: This work was supported by the National Natural Science Foundation of China, General Program (72573092 to QW), by the National Natural Science Foundation of China (72564016 to BG), and by Guangxi Philosophy and Social Science Research (25GLF134 to YP).
