# Supplementary material for: Unpacking the dual psychological paths of employee-AI collaboration on creativity: The role of proactive behavior
Source: PLoS One. 2026 Apr 24;21(4):e0347335. doi: 10.1371/journal.pone.0347335 (PMC13108763; doi:10.1371/journal.pone.0347335)
Supplement: S1 Table — (DOCX) [file pone.0347335.s001.docx]

S1 Table. Measurement Items

| Items of Employee-AI collaboration |
| --- |
| 1.AI participates in my decision-making process. |
| 2.AI participates in my prediction process. |
| 3.AI participates in my problem-solving process. |
| 4.AI participates in my information identification and evaluation process. |
| 5.AI participates in my problems, opportunities, or risk recognition process. |
| Items of Proactive behavior |
| 1.I actively attack problems. |
| 2.Whenever something goes wrong, I search for a solution immediately. |
| 3.Whenever there is a chance to get actively involved, I take it. |
| 4.I take initiative immediately even when others don’t. |
| 5.I use opportunities quickly in order to attain my goals. |
| 6.Usually, I do more work than what my supervisor requires. |
| 7.I am particularly good at realizing ideas. |
| Items of Self - efficacy |
| 1. If I try my best, I can always solve difficult problems. |
| 2. Even if others oppose me, I still find a way to get what I want. |
| 3. For me, upholding ideals and achieving goals is effortless. |
| 4. I am confident that I can effectively deal with any unexpected situation. |
| 5.With my intelligence, I can definitely deal with unexpected situations |
| 6. If I put in the necessary effort, I can definitely solve most difficult problems. |
| 7.I can face difficulties calmly because I can trust my ability to handle problems. |
| 8.When facing a difficult problem, I can usually find several solutions. |
| 9.When in trouble, I can usually think of some ways to cope. |
| 10.No matter what happens to me, I can handle it with ease. |
| Items of Performance pressure |
| 1.The performance pressure in my workplace is very high. |
| 2.I feel tremendous pressure to produce results. |
| 3.If I don’t produce at high levels, my job will be at risk. |
| 4.I would characterize my workplace as a results-driven environment. |
| Items of Creativity |
| 1.I take the initiative to try out new ideas or methods. |
| 2.Seeks new ideas and ways to solve problems. |
| 3.Generates ground-breaking ideas related to the field. |
| 4. Is a good role model for creativity. |
