# Supplementary material for: Unpacking the dual psychological paths of employee-AI collaboration on creativity: The role of proactive behavior
Source: PLoS One. 2026 Apr 24;21(4):e0347335. doi: 10.1371/journal.pone.0347335 (PMC13108763; doi:10.1371/journal.pone.0347335)
Supplement: S2 Table — (DOCX) [file pone.0347335.s002.docx]

S2 Table. The Latent Common-Method Factor Test

|  | X^2^/df | RMSEA | CFI | GFI | TLI |
| --- | --- | --- | --- | --- | --- |
| Original Model | 1.765 | 0.032 | 0.972 | 0.970 | 0.940 |
| Common Method Factor Model | 1.412 | 0.024 | 0.986 | 0.984 | 0.956 |
| Model Fit Change |  | ∆RMSEA | ∆CFI | ∆GFI | ∆TLI |
|  |  | 0.008 | 0.014 | 0.014 | 0.016 |
