# Supplementary material for: Unpacking the dual psychological paths of employee-AI collaboration on creativity: The role of proactive behavior
Source: PLoS One. 2026 Apr 24;21(4):e0347335. doi: 10.1371/journal.pone.0347335 (PMC13108763; doi:10.1371/journal.pone.0347335)
Supplement: S3 Table — (DOCX) [file pone.0347335.s003.docx]

S3 Table. Descriptive Analysis of Phase Two Samples

| Variables | Category | Number of people | Percentage | |
| --- | --- | --- | --- | --- |
|  |  |  | Two | One |
| Gender | Male | 125 | 53.9% | 50.3% |
|  | Female | 107 | 46.1% | 49.7% |
| Age | 21-30 years old | 91 | 39.2% | 38.1% |
|  | 31-40 years old | 94 | 40.5% | 31.2% |
|  | 41 years old and above | 47 | 20.3% | 30.7% |
| Education | College diploma or below | 76 | 32.8% | 36.3% |
|  | Bachelor's degree | 117 | 50.4% | 30.6% |
|  | Master's degree or above | 39 | 16.8% | 33.1% |
| Service tenure | Less than 2 years | 64 | 27.6% | 24.0% |
|  | 3-5 years | 84 | 36.2% | 35.9% |
|  | 6-10 years | 46 | 19.8% | 22.1% |
|  | 11 years and above | 38 | 16.4% | 18.0% |
| Position | Management | 48 | 20.7% | 12.2% |
|  | Technical | 62 | 26.7% | 24.8% |
|  | Research and Development | 47 | 20.3% | 23.8% |
|  | Service | 49 | 21.1% | 24.2% |
|  | Other | 26 | 11.2% | 15% |
