# Supplementary material for: Unpacking the dual psychological paths of employee-AI collaboration on creativity: The role of proactive behavior
Source: PLoS One. 2026 Apr 24;21(4):e0347335. doi: 10.1371/journal.pone.0347335 (PMC13108763; doi:10.1371/journal.pone.0347335)
Supplement: S4 Table — (DOCX) [file pone.0347335.s004.docx]

S4 Table. Data Path Analysis for Phase Two

| Variables | Creativity | | Self - efficacy | | Performance pressure | |
| --- | --- | --- | --- | --- | --- | --- |
|  | β | SE | β | SE | β | SE |
| Gender | -0.001 | 0.061 | -0.027 | 0.055 | -0.051 | 0.071 |
| Age | 0.015 | 0.041 | -0.038 | 0.037 | -0.053 | 0.047 |
| Education | -0.082 | 0.045 | 0.085^*^ | 0.040 | 0.045 | 0.051 |
| Service tenure | 0.022 | 0.030 | -0.024 | 0.027 | 0.091^**^ | 0.034 |
| Position | -0.015 | 0.024 | 0.016 | 0.022 | 0.062^*^ | 0.028 |
| Employee-AI collaboration | 0.280^***^ | 0.066 | 0.496^***^ | 0.056 | 0.397^***^ | 0.071 |
| Proactive behavior |  |  | 0.316^***^ | 0.068 | 0.459^***^ | 0.086 |
| Interaction term |  |  | 0.188^***^ | 0.041 | -0.122^*^ | 0.053 |
| Self - efficacy | 0.251^***^ | 0.070 |  |  | 0.293^***^ | 0.083 |
| Performance pressure | 0.194^***^ | 0.055 |  |  |  |  |
| R2 | 0.572 | | 0.673 | | 0.552 | |
