# Supplementary material for: Unpacking the dual psychological paths of employee-AI collaboration on creativity: The role of proactive behavior
Source: PLoS One. 2026 Apr 24;21(4):e0347335. doi: 10.1371/journal.pone.0347335 (PMC13108763; doi:10.1371/journal.pone.0347335)
Supplement: S5 Table — (DOCX) [file pone.0347335.s005.docx]

S5 Table. The Mediating Effect for Second Phase

| Path | β | SE | 95%CI | |
| --- | --- | --- | --- | --- |
|  |  |  | LB | UB |
| Employee-AI collaboration→Self-efficacy→Creativity | 0.172 | 0.072 | 0.041 | 0.326 |
| Employee-AI collaboration→Performance pressure→Creativity | 0.141 | 0.044 | 0.056 | 0.227 |
| Employee-AI collaboration→Self-efficacy→Performance pressure→Creativity | 0.039 | 0.019 | 0.009 | 0.082 |
