# Supplementary material for: Unpacking the dual psychological paths of employee-AI collaboration on creativity: The role of proactive behavior
Source: PLoS One. 2026 Apr 24;21(4):e0347335. doi: 10.1371/journal.pone.0347335 (PMC13108763; doi:10.1371/journal.pone.0347335)
Supplement: S6 Table — (DOCX) [file pone.0347335.s006.docx]

S6 Table. The Moderated Mediating Effect Test for Second Phase

| Moderator variable | Employee-AI collaboration→Self-efficacy→Creativity | | | Employee-AI collaboration→Performance pressure→Creativity | | |
| --- | --- | --- | --- | --- | --- | --- |
|  | β | SE | 95%CI | β | SE | 95%CI |
| Mean+1SD | 0.158 | 0.059 | [0.045,0.278] | 0.060 | 0.029 | [0.013,0.128] |
| Mean-1SD | 0.091 | 0.048 | [0.021,0.206] | 0.094 | 0.035 | [0.034,0.168] |
| Difference | 0.067 | 0.026 | [0.013,0.114] | -0.034 | 0.018 | [-0.070,-0.002] |
